# Draft Genome Sequence of the Nonpathogenic, Thermotolerant, and Exopolysaccharide-Producing *Bacillus anthracis* Strain PFAB2 from Panifala Hot Water Spring in West Bengal, India

**DOI:** 10.1128/genomeA.01346-16

**Published:** 2016-12-22

**Authors:** Aparna Banerjee, Urmi Halder, Vasvi Chaudhry, Rajeev K. Varshney, Shrikant Mantri, Rajib Bandopadhyay

**Affiliations:** a Department of Botany, UGC-Center of Advanced Study, The University of Burdwan, Golapbag, Bardhaman, West Bengal, India; b CSIR, Institute of Microbial Technology, Chandigarh, India; c Centre of Excellence in Genomics, International Crops Research Institute for the Semi-Arid Tropics (ICRISAT), Patancheru, India; d National Agri-Food Biotechnology Institute (NABI), S.A.S. Nagar, Mohali, Punjab, India

## Abstract

*Bacillus anthracis* is the causative agent of fatal anthrax in both animals and humans. It is prevalently pathogenic. Here, we present a *Bacillus anthracis* PFAB2 strain from a relatively unexplored Panifala hot water spring in West Bengal, India. It is nonpathogenic, exopolysaccharide producing, and thermotolerant in nature.

## GENOME ANNOUNCEMENT

Due to the large number of industrial applications of exopolysaccharides (EPSs), remarkable advancement has been observed in the discovery of new microbial EPSs (1). Among EPS producers, thermophiles comprise the least studied group, with very few reports despite their fast metabolism leading to better production (2, 3). *Bacillus anthracis* is the etiological agent of anthrax, causing fatal animal and human disease, and it was misused earlier as a biological weapon (4, 5). An *in silico* study suggested that *Bacillus anthracis* is going through progressive virulence gene loss into a nonpathogenic one (6).

Bacteria were isolated from a water sample from the Panifala hot water spring, West Bengal, India, in nutrient agar (NA) medium at 45°C overnight incubation. Whole-genome sequencing was done using an Illumina-MiSeq facility (ICRISAT, Patancheru, India). Before library construction, DNA quality and quantity were checked using a Qubit fluorometer (Thermo Fisher Scientific, USA). Libraries were constructed from 1 µg of genomic DNA using the Illumina TruSeq DNA PCR-free high-throughput (HT) library preparation kit (Illumina, USA). DNA was fragmented using Covaris AFA (Covaris, USA), followed by end repair and adapter ligation. Then, final libraries were denatured and sequenced on a MiSeq (Illumina).

The sequence data consist of 1,036,278 paired-end reads comprising 77,217,490 bases. Read quality was checked using the FastQC tool. CLC Assembly Cell was used for genome assembly, forming 390 contigs (*N*_50_, 29,785 bp), comprising 5,140,582 bases. The NCBI Prokaryotic Genome Annotation Pipeline (PGAP) (http://www.ncbi.nlm.nih.gov/genomes/static/Pipeline.html) and Rapid Annotations using Subsystems Technology (RAST) (7) were used to annotate genes of the draft genome sequence. The SEED viewer (8) was used to categorize predicted genes into functional subsystems.

The resulting draft genome sequence of *Bacillus anthracis* PFAB2 is 5,140,582 bp, with a mean G+C content of 35.1%. A total of 5,474 genes were annotated, out of which 5,147 genes were coding, with two rRNAs, six noncoding RNAs (ncRNAs), and 19 tRNAs. In RAST, 5,281 coding sequences were annotated, of which 45% (2,376) were assigned subsystems, while 55% (2905) had no match to any subsystem. Based on average nucleotide identity (ANI) analysis (http://www.ezbiocloud.net/tools/ani), PFAB2 showed 97.93% similarity with reference strain *Bacillus anthracis* strain Ames. Plasmid analysis was performed using the NCBI plasmid database, and interestingly, virulence plasmids harboring genes responsible for the pathogenicity characteristic of pathogenic *Bacillus anthracis* are absent in PBAB2.

Genome analysis revealed important genes responsible for exopolysaccharide synthesis: glycosyl transferase in contigs 34, 107, and 177, and EpsC and EpsD in contig 38. Also, a total of 17 stress-related heat shock proteins are present: GroEL and GroES in contig 293, Hsp20 in contigs 53 and 346, GrpE in contig 4, and chaperonin Hsp33 and ribosome-associated heat shock protein in contig 16. One important characteristic for virulence in pathogenic *Bacillus anthracis* is the presence of a poly-γ-d-glutamic acid capsule, which is vital for its survival in host, enabling it to evade the host immune system (9). In contrast, genes encoding poly-γ-glutamic acid biosynthesis are completely absent in PFAB2. All these features are probable indicators that *Bacillus anthracis* PFAB2 is thermotolerant in nature and a potent exopolysaccharide producer. The absence of important virulence-related marker genes is a feasible sign of its nonpathogenic nature. To our knowledge, this is the first report of a nonpathogenic, thermotolerant *Bacillus anthracis* strain from a hot water spring.

### Accession number(s).

The whole-genome sequence of *Bacillus anthracis* PFAB2 has been deposited at DDBJ/EMBL/GenBank under the accession no. MEAQ00000000.
